# George P. Rowell and Company, Publishers of the American Newspaper Directory

**Published:** 1892-10

**Authors:** 


### GEORGE P. ROWELL AND COMPANY, PUBLISHERS OF THE AMERICAN NEWSPAPER DIRECTORY.

---

We publish below a portion of a clipping from Messrs. Rowell and Company's *Printer's Ink*, which explains itself. When satisfied with the evidence Messrs. Rowell and Company promptly paid the forfeit.

It may not be amiss to state in this connection that, at least, for the three years now ending THE JOURNAL has told the absolute truth about its circulation. Not only were the reported number of copies printed, but the fact that we frequently retained scarcely enough copies for our files shows that we circulated them. A glance over our advertising pages will not reveal any evidence of our having lost by telling the truth. Lying about the circulation of a publication is common, and those who make the false statements do not seem to think there is any moral turpitude in such conduct. The publisher who bribes postoffice clerks to receipt him and make him pay for more pounds than he mails, and shows these receipts as proof of circulation, or the publisher who combines the receipts for several issues, and publishes them as covering one, or the publisher who makes any false statement, written, printed or oral, swindles the advertiser, whether he thinks so or not. We know what it costs to publish THE JOURNAL without financial loss, and we base our rates of advertising upon this knowledge. We shall continue to conduct THE JOURNAL honestly and truthfully, and shall use every effort to expose those in competition who are likely to injure us by false claims. We regret that we had to begin so near home and with a dead publication, but there was nothing personal in the matter; it was purely business.

The publishers of the American Newspaper Directory have paid for the second time this year the $100 offered to any one who can prove to be incorrect a rating printed in Arabic figures. The case is that of the *Dixie Doctor*, of Atlanta, Ga., which was credited with 5,500 circulation. The paper is reported to have suspended publication some time ago. Dr. M. B Hutchins, one of the editors of THE MEDICAL AND SURGICAL JOURNAL offered the affidavit of E. M. Evans, a sub-foreman in the job office of the Atlanta *Constitution*, where the *Dixie Doctor* was printed, averring that the number of copies printed for the entire year was only 6,600.

